# Mediation of Racial and Ethnic Inequities in the Diagnosis of Advanced-Stage Cervical Cancer by Insurance Status

**DOI:** 10.1001/jamanetworkopen.2023.2985

**Published:** 2023-03-01

**Authors:** Hunter K. Holt, Caryn E. Peterson, Shannon MacLaughlan David, Abdullah Abdelaziz, George F. Sawaya, Jenny S. Guadamuz, Gregory S. Calip

**Affiliations:** Department of Family and Community Medicine, University of Illinois at Chicago, Chicago; Department of Epidemiology and Biostatistics, University of Illinois at Chicago, Chicago; Department of Obstetrics and Gynecology, University of Illinois at Chicago, Chicago; Department of Pharmacy Systems, Outcomes and Policy, University of Illinois at Chicago, Chicago; Department of Obstetrics, Gynecology, and Reproductive Sciences, University of California, San Francisco; Flatiron Health, New York, New York, Program on Medicines and Public Health, University of Southern California, Los Angeles; Department of Pharmacy Systems, Outcomes and Policy, University of Illinois at Chicago, Chicago, Flatiron Health, New York, New York

## Abstract

**IMPORTANCE:**

Black and Hispanic or Latina women are more likely than White women to receive a diagnosis of and to die of cervical cancer. Health insurance coverage is associated with diagnosis at an earlier stage of cervical cancer.

**OBJECTIVE:**

To evaluate the extent to which racial and ethnic differences in the diagnosis of advanced-stage cervical cancer are mediated by insurance status.

**DESIGN, SETTING, AND PARTICIPANTS:**

This retrospective, cross-sectional population-based study used data from the Surveillance, Epidemiology, and End Results (SEER) program on an analytic cohort of 23 942 women aged 21 to 64 years who received a diagnosis of cervical cancer between January 1, 2007, and December 31, 2016. Statistical analysis was performed from February 24, 2022, to January 18, 2023.

**EXPOSURES:**

Health inusurance status (private or Medicare insurance vs Medicaid or uninsured).

**MAIN OUTCOMES AND MEASURES:**

The primary outcome was a diagnosis of advanced-stage cervical cancer (regional or distant stage). Mediation analyses were performed to assess the proportion of observed racial and ethnic differences in the stage at diagnosis that were mediated by health insurance status.

**RESULTS:**

A total of 23 942 women (median age at diagnosis, 45 years [IQR, 37–54 years]; 12.9% were Black, 24.5% were Hispanic or Latina, and 52.9% were White) were included in the study. A total of 59.4% of the cohort had private or Medicare insurance. Compared with White women, patients of all other racial and ethnic groups had a lower proportion with a diagnosis of early-stage cervical cancer (localized) (American Indian or Alaska Native, 48.7%; Asian or Pacific Islander, 49.9%; Black, 41.7%; Hispanic or Latina, 51.6%; and White, 53.3%). A larger proportion of women with private or Medicare insurance compared with women with Medicaid or uninsured received a diagnosis of an early-stage cancer (57.8% [8082 of 13 964] vs 41.1% [3916 of 9528]). In models adjusting for age, year of diagnosis, histologic type, area-level socioeconomic status, and insurance status, Black women had higher odds of receiving a diagnosis of advanced-stage cervical cancer compared with White women (odds ratio, 1.18 [95% CI, 1.08–1.29]). Health insurance was associated with mediation of more than half (ranging from 51.3% [95% CI, 51.0%−51.6%] for Black women to 55.1% [95% CI, 53.9%−56.3%] for Hispanic or Latina women) the racial and ethnic inequities in the diagnosis of advanced-stage cervical cancer across all racial and ethnic minority groups compared with White women.

**CONCLUSIONS AND RELEVANCE:**

This cross-sectional study of SEER data suggests that insurance status was a substantial mediator of racial and ethnic inequities in advanced-stage cervical cancer diagnoses. Expanding access to care and improving the quality of services rendered for uninsured patients and those covered by Medicaid may mitigate the known inequities in cervical cancer diagnosis and related outcomes.

## Introduction

Cervical cancer incidence and mortality have steadily decreased since the introduction of cervical cytology (Papanicolaou tests) for screening.^1^ Although the main effect of screening is identification and treatment of cervical precancerous lesions, screening also identifies cancers. Individuals who receive a diagnosis of early-stage (localized) cervical cancer have over a 90% 5-year survival rate, while those who receive a diagnosis of more advanced-stage cancers have much lower 5-year survival rates (59% for regional cancers and 17% for distant or metastatic cancers).^1^ Despite advances in prevention, screening, and treatment of cervical cancer over the past few decades, Black, Hispanic or Latina, and American Indian or Alaska Native women in the US are more likely to receive a diagnosis of cervical cancer and more likely to receive a diagnosis of a more advanced stage of cervical cancer than White women.^2–8^ Furthermore, Black women are more likely than White women to receive inadequate treatment for and die of cervical cancer.^2,6,9^

Previous studies have found that cervical cancer outcomes, including stage at diagnosis and survival, are associated with insurance status.^2,9,10^ However, these studies did not evaluate insurance status as a mediator for more advanced cervical cancer stage at diagnosis between different racial and ethnic groups. Insurance status, treated as a mediator, can help quantify and explain the association of various racial and ethnic groups with diagnosis of advanced-stage cervical cancer.^11^ The objective of this study was to evaluate the extent to which health insurance coverage mediated advanced-stage cervical cancer diagnosis among various racial and ethnic groups in the US.

## Methods

### Selection and Description of Study Participants

This study is a retrospective, population-based, cross-sectional study that used data from the Surveillance, Epidemiology, and End Results (SEER) Program on patients who received a diagnosis of cervical cancer between January 1, 2007, and December 31, 2016 (the most recent year available). For the purpose of this study, we used the SEER Census Tract-level Socioeconomic Status (SES) and Rurality Database.^12^ The SEER program is funded by the National Institutes of Health National Cancer Institute and includes population-based cancer incidence data. The cancer diagnoses were identified using the *International Classification of Diseases for Oncology, Third Edition* (eTable 1 in Supplement 1). This specific database includes demographic information that allows for socioeconomic quintiles to be calculated, rurality, and clinical information, including a broader definition of staging (localized, regional, and distant), the American Joint Committee on Cancer (AJCC) stage, and treatment modalities. The University of Illinois at Chicago institutional review board reviewed this study and waived approval and informed consent, determining that it did not involve human participants research. This study followed the Strengthening the Reporting of Observational Studies in Epidemiology (STROBE) reporting guideline.

Women aged 21 to 64 years with a diagnosis of localized, regional, or distant cervical cancer were included in our study (n = 24 945). Our analytic cohort excluded women with an unknown exposure variable (unknown or other race and ethnicity [n = 246]), a mediator variable (unknown health insurance status [n = 909]), and/or an outcome variable (unknown stage of cervical cancer [n = 630]). We also excluded those with a cervical cancer diagnosis by autopsy, per death certificate only, and/or nonmicroscopically confirmed cases. We also excluded patients with a diagnosis of multiple cancers and a histologic subtype suggestive of another primary site (eTable 2 in Supplement 1). Our final analytic cohort included 23 492 individuals.

### Data Collection

Demographic information, including race and ethnicity, was collected at the time of cervical cancer diagnosis from SEER registry data. Our primary exposure of interest was race and ethnicity, which was coded as Hispanic or Latina (all races), non-Hispanic American Indian or Alaska Native (hereafter American Indian or Alaska Native), non-Hispanic Asian or Pacific Islander (hereafter Asian or Pacific Islander), non-Hispanic Black (hereafter Black), and non-Hispanic White (hereafter White). We also collected information on SEER summary diagnosis stage (localized, regional, or distant), AJCC stage, treatment modalities received (surgery, chemotherapy, and radiotherapy), and histologic subtype (squamous cell carcinoma, adenocarcinoma, adenosquamous carcinoma, and other) (eTable 1 in Supplement 1). Area-level variables included time-dependent SES (Yost SES-quintile, census tract-level) and rurality (US Department of Agriculture’s Rural Urban Commuting Area codes, county-level), which were collected at the time of diagnosis and based on the patient’s address.^12^

The outcome variable of interest for this study was advanced-stage cervical cancer, which we coded dichotomously based on the SEER summary stage (early-stage: localized SEER summary stage; advanced-stage: regional and distant SEER summary stage). Regional and distant summary stages were combined due to the large descrease in survivability.^1,6^

The mediator variable of interest was insurance status. The insurance recode variable, a SEER defined variable, was used. We specifically collected information and coded for uninsured individuals, those with Medicaid (which includes Indian or Public Health service), and insured individuals (which includes private insurance, Medicare, and military coverage).^13^ We were unable to ascertain duration of Medicaid coverage in the SEER data; thus, we combined uninsured women and those with Medicaid coverage at the time of diagnosis into 1 group for the purpose of analysis. This is important because there are programs that provide insurance coverage at the time of cancer diagnosis. For example, the Centers for Disease Control and Prevention National Breast and Cervical Cancer Early Detection Program provides Medicaid coverage for cancer treatment to uninsured women with a diagnosis of cervical cancer.^14^

### Statistical Analysis

Statistical analysis was performed from February 24, 2022, to January 18, 2023. We compared the demographic and clinical characteristics of patients by disease stage and race and ethnicity using descriptive statistics. To test for differences between groups, we used the Wilcoxon rank-sum test for median values, *Z*-test for proportions, and the χ^2^ test for categorical variables. Similar to previous reports using mediation methods to investigate cancer health inequities,^15,16^ we applied the product method approach proposed by Baron and Kenny^17^ and VanderWeele.^18,19^ In brief, we estimated the presence of mediation and direct and indirect effects using a single mediator model; this was accomplished through a series of regressions by conducting univariable and multivariable analyses of the primary exposure (ie, race and ethnicity) and a priori measured confounders (age, year of diagnosis, and histologic subtype) on the primary outcome variable of advanced-stage cervical cancer (ie, regional or distant stage cervical cancer). We then separately conducted univariable analysis and multivariable analysis, regressing the mediator of interest (ie, health insurance status) on race and ethnicity and a priori measured confounders (age, year of diagnosis, and histologic subtype). With these calculations, the natural direct effects, natural indirect effects, and total effects were estimated.^19^ Next, we calculated the extent that the exposure-outcome association was associated with the mediator by conducting a proportional mediated calculation using the PARAMED module in Stata,^20^ release 17 (StataCorp LLC). From the PARAMED module, we used the calculated coefficients from the logistic regression model to define the outcome variables and the calculated coefficients from the logistic regression model to characterize the mediator variables. Interaction from area-level SES with insurance status was accounted for by including this variable in the analysis. We calculated the variance and constructed the 95% CI estimates using the bootstrap method to account for the product method estimator being nonnormal.^21,22^ Finally, sensitivity analyses were performed to evaluate the proportion of inequity mediated by uninsured status compared with insured status, excluding patients who had Medicaid coverage, and patients with Medicaid insurance coverage compared with privately insured and Medicare-insured patients, excluding uninsured patients. All *P* values were from 2-sided tests, and results were deemed statistically significant at *P* < .05.

## Results

Our study included 23 492 women with a diagnosis of either localized, regional, or distant cervical cancer (Table 1). The median age at diagnosis for all stages of cancer was 45 years (IQR, 37–53 years). More than half (52.9%) the population was identified as White, 12.8% as Black, 0.8% as American Indian or Alaska Native, 9.0% as Asian or Pacific Islander, and 24.5% as Hispanic or Latina. Approximately two-thirds (67.1%) had a diagnosis of squamous cell carcinoma, 26.2% had a diagnosis of adenocarcinoma, 4.0% had a diagnosis of adenosquamous carcinoma, and 2.8% had a diagnosis of other types of cancer. Most women (59.4%) had insurance, while 8.0% were uninsured and 32.5% were insured by Medicaid. Individuals with private or Medicare insurance compared with those either without insurance or with Medicaid insurance were more likely to be diagnosed with an early-stage (localized) cancer (57.8% [8082 of 13 964] vs 41.1% [3916 of 9528]; *P* < .001).

A lower proportion of Black (41.7%), American Indian or Alaska Native (48.7%), Asian or Pacific Islander (49.9%), and Hispanic or Latina (51.6%) women received a diagnosis of early-stage cervical cancer (localized) compared with White Women (53.3%) (*P* < .001) (Table 2). In addition, more White women had private or Medicare insurance at the time of diagnosis (69.4%) compared with all the other racial and ethnic groups in the study (Black, 48.2%; American Indian or Alaska Native, 48.7%; Asian or Pacific Islander, 64.3%; and Hispanic or Latina, 42.5%; *P* < .001).

A larger proportion of White women than Black women underwent surgery for early-stage cervical cancer (localized) (91.1% vs 82.8%; *P* < .001) (eTable 3 in Supplement 1). This trend continued for more advanced-stage (regional or distant) cancers, with 33.9% of White women and 24.3% of Black women undergoing surgery (*P* < .001).

In the multivariable regression analysis, we found that Black women and Hispanic or Latina women had a higher odds of diagnosis with advanced-stage cervical cancer compared with White women, after adjustment for age, year of diagnosis, and histologic subtype (Black: adjusted odds ratio [aOR], 1.39 [95% CI, 1.27–1.51]; Hispanic or Latina: OR, 1.13 [95% CI, 1.06–1.21]) (Table 3). These associations were attenuated when the models included area-level SES and insurance status for Black women (aOR, 1.18 [95% CI, 1.08–1.29]) and were reversed for Hispanic or Latina women (aOR, 0.93 [95% CI, 0.87–0.99]). In our second analysis, after adjusting for age, year of diagnosis, and area-level SES, all racial and ethnic groups had statistically significant increased odds of being uninsured or having Medicaid insurance at the time of cervical cancer diagnosis compared with White women (Black: aOR, 1.64 [95% CI, 1.50–1.79]; American Indian or Alaska Native: aOR, 1.98 [95% CI, 1.48–2.67]; Asian or Pacific Islander: aOR, 1.44 [95% CI, 1.30–1.59]; and Hispanic or Latina: aOR, 2.54 [95% CI, 2.37–2.71]) (Table 4).

In the mediation analysis, we found that being uninsured or insured by Medicaid accounted for more than half (ranging from 51.3% [95% CI, 51.0%−51.6%] for Black women to 55.1% [95% CI, 53.9%−56.3%] for Hispanic or Latina women) the estimated inequity in advanced-stage (regional or distant) cervical cancer diagnoses across racial and ethnic groups compared with White women (Table 5). Sensitivity analyses evaluating uninsured vs insured status and Medicaid insurance vs private or Medicare insurance status found similar proportions mediated across all racial and ethnic groups compared with White women (eTable 4 in Supplement 1).

## Discussion

Our study confirmed previous findings that, compared with White women, Black women, as well as women from all other racial and ethnic groups included in the study, were more likely to receive a diagnosis of advanced-stage cervical cancer.^23–28^ We also found that insurance status mediated more than half of the inequities in the diagnosis of advanced-stage cervical cancer among all other racial and ethnic groups compared with White women. This finding could explain known differences in the stage of diagnosis for different racial and ethnic groups. Although previous studies have found inequities associated with health insurance and stage of cervical cancer,^10,28,29^ we quantify the association of insurance status with the diagnosis of more advanced-stage cancer by using population-based data and formally treating insurance status as a mediator. Health insurance status is a modifiable risk factor that could reduce these persistent cervical cancer mortality inequities among Black individuals.^30^ Extrapolating our results, even among populations that do not possess differences in cervical cancer mortality, far fewer individuals of all races and ethnicities would receive a diagnosis of advanced-stage cancer and, thus, would have a higher 5-year survival rate.

Despite the Patient Protection and Affordable Care Act and programs such as the Centers for Disease Control and Prevention’s National Breast and Cervical Cancer Early Detection Program, there is an increasing proportion of the population that has not received up-to-date cervical cancer screening services.^31,32^ Uninsured individuals and those with Medicaid insurance are the most likely to be underscreened.^31–33^ Even if these populations receive screening, both uninsured individuals and individuals with Medicaid are more likely to experience delays in accessing the following diagnostic procedures necessary to diagnose actual cancer.^28,34–36^ This lack of access to initial screening and barriers to follow-up care could explain why, in racial and ethnic minority groups, women who are uninsured or covered by Medicaid present with more cases of advanced-stage cervical cancer.

These findings are important in the context of previous studies investigating the survivability of patients from racial and ethnic groups after a diagnosis of cervical cancer. One study found that insurance status mediated less than 19% of the association with excess mortality among Black women compared with White women.^2^ That study also found that a more important mediator of survivability was the stage of diagnosis. In context with our findings, if private or Medicare insurance coverage was expanded or if Medicaid eased the administrative burden physicians face,^37^ far fewer women from racial and ethnic minority groups would receive a diagnosis of advanced-stage cancer and, thus, would have a higher survivability.

Although this study found that insurance status is a significant mediator of the racial and ethnic inequities in advanced-stage cervical cancer diagnosis, there are numerous additional modifiable factors that play a major role in the prevention of cancer. Societal, community, and individual factors are associated with differential uptake of cervical cancer screening services and affect the ability to follow up after an abnormal test result.^36^ Stigma related to human papillomavirus infection or inability to receive screening can prevent further follow-up care,^38^ which can result in more advanced-stage cancer diagnoses. Also, human papillomavirus vaccination is an effective primary prevention intervention, and efforts should focus on ensuring that all eligible populations are vaccinated.^39^

Nevertheless, this study adds to the growing literature that highlights the poor outcomes for individuals who are underinsured.^2,9,15,40^ Future policy changes need to focus on expanding access to care and improving the quality of services rendered for uninsured patients and those covered by Medicaid to specifically address this cancer inequity for historically marginalized racial and ethnic groups. Furthermore, to reduce inequities in the incidence of cervical cancer and in mortality and achieve the World Health Organization’s mandate to eliminate the disease, a multimodal approach is necessary to address all modifiable factors. Adopting a multifactorial approach that integrates clinical and financial care navigation to improve access to health care services,^41^ eliminates racism in medicine or health care,^42–44^ and adapts a socioecological model to address social determinants of health that prevent access to health care^44,45^ will be necessary to fully address these cervical cancer inequities.

### Limitations

This study has several limitations. First, the population sampled for our analysis may not be generalizable to the whole population of the US.^46^ Second, insurance information may be flawed, especially as patients who initially received a diagnosis of cervical cancer may have enrolled in Medicaid by the time they were enrolled in the registry data,^47,48^ potentially underestimating the effectiveness of Medicaid insurance coverage at preventing advanced-stage cervical cancer. In addition, SEER only reports insurance status data up to 2016; thus, more recent analysis investigating insurance status and disease stage at diagnosis of cervical cancer is not possible at this time.^13^ Third, while SEER has AJCC staging information, there is a higher frequency of missing AJCC stage data. In comparison, SEER has much more robust data for the 3 summary stages: localized, regional, and distant. This may limit the specificity of our findings due to the large grouping of the AJCC and International Federation of Gynecology and Obstetrics stages within these 3 categories. Fourth, SEER lacks data regarding cervical precancers; this limits our ability to conduct further analysis comparing precancer and early-stage cancer. Future studies could consider evaluating insurance status and odds of precancerous diagnosis compared with cancer diagnosis. Fifth, the analysis was able to account for only area-level SES; future studies using individual-level SES could provide more insight into the odds of developing advanced-stage cancer. Sixth, SEER data on race and ethnicity are coded using their underlying cancer registry, which has a history of misclassifications of race and ethnicity compared with the criterion standard of self-report.^49,50^ Future efforts to improve the accuracy of the information we collect, especially regarding race and ethnicity, is critical for successful health equity research.^51^

## Conclusions

Our study found that insurance status mediated more than half of the advanced-stage cervical cancers diagnosed among women from racial and ethnic minority groups. Although our findings suggest that a large proportion of the cancer inequities was associated with insurance status, we also acknowledge that equity in insurance coverage will not eliminate cervical cancer unilaterally.

## Supplementary Material

Supplemental Material **eTable 1.** Inclusion and Classification of Cancer Subtype *ICD-O-3*
**eTable 2.** Exclusion *ICD-O-3* Codes
**eTable 3.** Rates of Treatment With Surgery, Radiation, and Chemotherapy Among Women With Cervical Cancer by Race/Ethnicity and Summary Stage
**eTable 4.** Multivariable Product Method Estimates for Proportion of Stage-Related Inequities Mediated by Insurance Status Across Racial/Ethnic Groups

Supplemental Material 2 Data Sharing Statement

## Figures and Tables

**Table 1.** Descriptive Characteristics of Patients With Cervical Cancer by Surveillance, Epidemiology, and End Results Program Summary Stage

Characteristic	Women, No. (%) All (N = 23 492)			*P* value^a^
	Women, No. (%) All (N = 23 492)	Localized cancer (n = 11 998)	Regional and distant cancer (n = 11 494)	
Age, y				
Median (IQR)	45 (37–53)	42 (35–50)	48 (40–56)	<.001
<30	1565 (6.7)	1066 (8.9)	499 (4.3)	<.001
30–49	13 601 (57.9)	7891 (65.8)	5710 (49.7)	
50–64	8326 (35.4)	3041 (25.3)	5285 (46.0)	
Year of diagnosis				
2007–2009	7188 (30.6)	3789 (31.6)	3399 (29.6)	.003
2010–2012	6940 (29.5)	3519 (29.3)	3421 (29.8)	
2013–2016	9364 (39.9)	4690 (39.1)	4674 (40.7)	
Histologic subtype				
Squamous	15 760 (67.1)	7252 (60.4)	8508 (74.0)	<.001
Adenocarcinoma	6156 (26.2)	4062 (33.9)	2094 (18.2)	
Adenosquamous	929 (4.0)	446 (3.7)	483 (4.2)	
Other	647 (2.8)	238 (2.0)	409 (3.6)	
Race and ethnicity				
American Indian or Alaska Native	187 (0.8)	91 (0.8)	96 (0.8)	<.001
Asian or Pacific Islander	2108 (9.0)	1052 (8.8)	1056 (9.2)	
Black	3017 (12.8)	1258 (10.5)	1759 (15.3)	
Hispanic or Latina	5745 (24.5)	2966 (24.7)	2779 (24.2)	
White	12 435 (52.9)	6631 (55.3)	5804 (50.5)	
Surgery				
Yes	14 484 (61.7)	10 758 (89.7)	3726 (32.4)	<.001
No	9008 (38.3)	1240 (10.3)	7768 (67.6)	
Radiotherapy				
Yes	12 597 (53.6)	2959 (24.7)	9638 (83.9)	<.001
No or unknown	10 895 (46.4)	9039 (75.3)	1856 (16.1)	
Chemotherapy				
Yes	11 605 (49.4)	2184 (18.2)	9421 (82.0)	<.001
No or unknown	11 887 (50.6)	9814 (81.8)	2073 (18.0)	
Insurance status				
Uninsured	1882 (8.0)	751 (6.3)	1131 (9.8)	<.001
Any Medicaid	7646 (32.5)	3165 (26.4)	4481 (39.0)	
Insured	13 964 (59.4)	8082 (67.4)	5882 (51.2)	
Marital status				
Married	10 508 (44.7)	5870 (48.9)	4638 (40.4)	<.001
Not married	11 941 (50.8)	5530 (46.1)	6411 (55.8)	
Unknown	1043 (4.4)	598 (5.0)	445 (3.9)	
Yost SES quintile				
First (lowest SES)	5852 (24.9)	2652 (22.1)	3200 (27.8)	<.001
Second	5007 (21.3)	2414 (20.1)	2593 (22.6)	
Third	4328 (18.4)	2204 (18.4)	2124 (18.5)	
Fourth	3899 (16.6)	2192 (18.3)	1707 (14.9)	
Fifth (highest SES)	3159 (13.4)	1875 (15.6)	1284 (11.2)	
Unknown	1247 (5.3)	661 (5.5)	586 (5.1)	
RUCA category				
Urban	20 909 (89.0)	10 725 (89.4)	10 184 (88.6)	.03
Rural	1619 (6.9)	775 (6.5)	844 (7.3)	
Unknown	964 (4.1)	498 (4.2)	466 (4.1)	

Abbreviations: RUCA, rural-urban continuum code; SES, socioeconomic status.

a To test for differences between groups, we used the Wilcoxon rank sum test for median values and the χ^2^ test for categorical variables.

**Table 2.** Descriptive Characteristics of Patients With Cervical Cancer by Race and Ethnicity

Characteristic	Women, No. (%)					*P* value^a^
	Hispanic or Latina (n = 5745)	American Indian or Alaska Native (n = 187)	Asian or Pacific Islander (n = 2108)	Black (n = 3017)	White (n = 12 435)	
Age, y						
Median (IQR)	43 (36–51)	44 (37–51)	47 (39–55)	46 (38–54)	45 (37–54)	<.001
<30	444 (7.7)	10 (5.3)	83 (3.9)	201 (6.7)	827 (6.7)	
30–49	3624 (63.1)	123 (65.8)	1161 (55.1)	1611 (53.4)	7082 (57.0)	
50–64	1677 (29.2)	54 (28.9)	864 (41.0)	1205 (39.9)	4526 (36.4)	
Year of diagnosis						
2007–2009	1743 (30.3)	46 (24.6)	602 (28.6)	946 (31.4)	3851 (31.0)	.04
2010–2012	1660 (28.9)	62 (33.2)	611 (29.0)	905 (30.0)	302 (2.4)	
2013–2016	2342 (40.8)	79 (42.2)	895 (42.5)	1166 (38.6)	4882 (39.3)	
Histologic subtype						
Squamous	3878 (67.5)	131 (70.1)	1335 (63.3)	2465 (81.7)	7951 (63.9)	<.001
Adenocarcinoma	1471 (25.6)	44 (23.5)	599 (28.4)	363 (12.0)	3679 (29.6)	
Adenosquamous	238 (4.1)	9 (4.8)	112 (5.3)	92 (3.0)	478 (3.8)	
Other	158 (2.8)	3 (1.6)	62 (2.9)	97 (3.2)	327 (2.6)	
SEER summary stage						
Localized	2966 (51.6)	91 (48.7)	1052 (49.9)	1258 (41.7)	6631 (53.3)	<.001
Regional	2122 (36.9)	67 (35.8)	801 (38.0)	1258 (41.7)	4169 (33.5)	
Distant	657 (11.4)	29 (15.5)	255 (12.1)	501 (16.6)	1635 (13.1)	
Surgery						
Yes	3546 (61.7)	111 (59.4)	1351 (64.1)	1469 (48.7)	8007 (64.4)	<.001
No	2199 (38.3)	76 (40.6)	757 (35.9)	1548 (51.3)	4428 (35.6)	
Radiotherapy						
Yes	3063 (53.3)	102 (54.5)	1090 (51.7)	1890 (62.6)	6452 (51.9)	<.001
No or unknown	2682 (46.7)	85 (45.5)	1018 (48.3)	1127 (37.4)	5983 (48.1)	
Chemotherapy						
Yes	2818 (49.1)	102 (54.5)	1023 (48.5)	1707 (56.6)	5955 (47.9)	<.001
No or unknown	2927 (50.9)	85 (45.5)	1085 (51.5)	1310 (43.4)	6480 (52.1)	
Insurance status						
Uninsured	700 (12.2)	3 (1.6)	116 (5.5)	341 (11.3)	722 (5.8)	<.001
Any Medicaid	2606 (45.4)	93 (49.7)	637 (30.2)	1223 (40.5)	3087 (24.8)	
Insured	2439 (42.5)	91 (48.7)	1355 (64.3)	1453 (48.2)	8626 (69.4)	
Marital status						
Married	2440 (42.5)	61 (32.6)	1249 (59.3)	701 (23.2)	6057 (48.7)	<.001
Not married	3065 (53.4)	118 (63.1)	788 (37.4)	2162 (71.7)	5808 (46.7)	
Unknown	240 (4.2)	8 (4.3)	71 (3.4)	154 (5.1)	570 (4.6)	
Yost SES quintile						
First (lowest SES)	1917 (33.4)	40 (21.4)	269 (12.8)	1557 (51.6)	2069 (16.6)	<.001
Second	1476 (25.7)	52 (27.8)	336 (15.9)	622 (20.6)	2521 (20.3)	
Third	984 (17.1)	33 (17.6)	388 (18.4)	379 (12.6)	2544 (20.5)	
Fourth	686 (11.9)	17 (9.1)	531 (25.2)	243 (8.1)	2422 (19.5)	
Fifth (highest SES)	413 (7.2)	7 (3.7)	491 (23.3)	104 (3.4)	2144 (17.2)	
Unknown	269 (4.7)	38 (20.3)	93 (4.4)	112 (3.7)	735 (5.9)	
RUCA category						
Urban	5400 (94.0)	120 (64.2)	1998 (94.8)	2792 (92.5)	10 599 (85.2)	<.001
Rural	134 (2.3)	29 (15.5)	56 (2.7)	151 (5.0)	1249 (10.0)	
Unknown	211 (3.7)	38 (20.3)	54 (2.6)	74 (2.5)	587 (4.7)	

Abbreviations: RUCA, rural-urban continuum code; SEER, Surveillance, Epidemiology, and End Results Program; SES, socioeconomic status.

a To test for differences between groups, we used the Wilcoxon rank sum test for median values and the χ^2^ test for categorical variables.

**Table 3.** Multivariable Logistic Regression Analyses for Associations Between Race and Ethnicity and Diagnosis With Regional or Distant Cervical Cancer

Race and ethnicity	Unadjusted model		Confounder-adjusted model^a^		Confounder- and mediator-adjusted model^b^	
	OR (95% CI)	*P* value	OR (95% CI)	*P* value	OR (95% CI)	*P* value
American Indian or Alaska Native	1.21 (0.90–1.61)	.21	1.22 (0.91–1.65)	.19	1.07 (0.79–1.45)	.66
Asian or Pacific Islander	1.15 (1.05–1.26)	.01	1.07 (0.97–1.17)	.20	1.06 (0.96–1.17)	.25
Black	1.60 (1.47–1.73)	<.001	1.39 (1.27–1.51)	<.001	1.18 (1.08–1.29)	<.001
Hispanic or Latina	1.07 (1.01–1.14)	.03	1.13 (1.06–1.21)	<.001	0.93 (0.87–0.99)	.04
White	1 [Reference]	NA	1 [Reference]	NA	1 [Reference]	NA

Abbreviations: NA, not applicable; OR, odds ratio.

a Included multivariable adjustment for age, year of diagnosis, and histologic subtype.

b Included multivariable adjustment for age, year of diagnosis, histologic subtype, insurance status, and analysis with area-level socioeconomic status as an interaction variable.

**Table 4.** Multivariable Logistic Regression Analyses for Associations Between Race and Ethnicity and Being Uninsured or Having Medicaid Coverage

Race and ethnicity	Unadjusted model		Confounder-adjusted model^a^		Confounder- and mediator-adjusted model^b^	
	OR (95% CI)	*P* value	OR (95% CI)	*P* value	OR (95% CI)	*P* value
American Indian or Alaska Native	2.39 (1.79–3.19)	<.001	2.41 (1.80–3.21)	<.001	1.98 (1.48–2.67)	<.001
Asian or Pacific Islander	1.26 (1.14–1.39)	<.001	1.25 (1.14–1.38)	<.001	1.44 (1.30–1.59)	<.001
Black	2.44 (2.25–2.64)	<.001	2.43 (2.24–2.64)	<.001	1.64 (1.50–1.79)	<.001
Hispanic or Latina	3.07 (2.88–3.27)	<.001	3.10 (2.90–3.31)	<.001	2.54 (2.37–2.71)	<.001
White	1 [Reference]	NA	1 [Reference]	NA	1 [Reference]	NA

Abbreviations: NA, not applicable; OR, odds ratio.

a Included multivariable adjustment for age and year of diagnosis.

b Included multivariable adjustment for age, year of diagnosis, and analysis with area-level socioeconomic status as an interaction variable.

**Table 5.** Multivariable Product Method Estimates for Proportion of Stage-Related Inequities Mediated by Insurance Status Across Racial and Ethnic Groups^a^

Race and ethnicity	% Mediated (95% CI)
American Indian or Alaska Native	52.5 (51.9–53.2)
Asian or Pacific Islander	53.8 (52.9–54.7)
Black	51.3 (51.0–51.6)
Hispanic or Latina	55.1 (53.9–56.3)
White	[Reference]

a Estimates including area-level socioeconomic status as an interaction term were similar to our primary approach; results presented here incorporate area-level socioeconomic status as an interaction variable of the observed racial and ethnic inequities.

## Key Points

### Question

How much of the racial and ethnic differences in diagnosis of advanced-stage (regional or distant stage) cervical cancer is mediated by insurance status?

### Findings

This cross-sectional study of 23 942 individuals found that more than half of the diagnoses of advanced-stage cervical cancer among individuals from minority racial and ethnic groups were mediated by insurance coverage or type.

### Meaning

This study suggests that insurance is a modifiable risk factor that plays an important role in the racial and ethnic inequities observed in the diagnosis of advanced-stage cervical cancer.
