# Supplementary material for: Mediation of Racial and Ethnic Inequities in the Diagnosis of Advanced-Stage Cervical Cancer by Insurance Status
Source: JAMA Netw Open. Author manuscript; Available in PMC 2023 Dec 18. (PMC10726717; doi:10.1001/jamanetworkopen.2023.2985)
Supplement: Supplemental Material — eTable 1. Inclusion and Classification of Cancer Subtype ICD-O-3 eTable 2. Exclusion ICD-O-3 Codes eTable 3. Rates of Treatment With Surgery, Radiation, and Chemotherapy Among Women With Cervical Cancer by Race/Ethnicity and Summary Stage eTable 4. Multivariable Product Method Estimates for Proportion of Stage-Related Inequities Mediated by Insurance Status Across Racial/Ethnic Groups [file NIHMS1943084-supplement-Supplemental_Material.pdf]

Supplementary Online Content

Holt HK, Peterson CE, MacLaughlan David S, et al. Mediation of racial and ethnic inequities in the diagnosis of advanced-stage cervical cancer by insurance status. *JAMA Netw Open.* 2023;6(3):e232985. doi:10.1001/jamanetworkopen.2023.2985

eTable 1. Inclusion and Classification of Cancer Subtype *ICD-O-3*
eTable 2. Exclusion *ICD-O-3* Codes
eTable 3. Rates of Treatment With Surgery, Radiation, and Chemotherapy Among Women With Cervical Cancer by Race/Ethnicity and Summary Stage
eTable 4. Multivariable Product Method Estimates for Proportion of Stage-Related Inequities Mediated by Insurance Status Across Racial/Ethnic Groups

This supplementary material has been provided by the authors to give readers additional information about their work.

© 2023 Holt HK et al. *JAMA Network Open.*

eTable 1. Inclusion and Classification of Cancer Subtype *ICD-O-3*

SEER ICD-O 3 Histology Code	Type	Histology Classification
8050	Papilloma (NOS)	Squamous Cell Carcinoma
8051	Verrucous carcinoma	Squamous Cell Carcinoma
8052	Squamous cell papilloma	Squamous Cell Carcinoma
8070	Squamous cell carcinoma NOS	Squamous Cell Carcinoma
8071	Squamous cell Carcinoma, keratinizing	Squamous Cell Carcinoma
8072	Squamous cell carcinoma, large cell, non-keratinizing	Squamous Cell Carcinoma
8073	Squamous cell carcinoma, small cell, non-keratinizing	Squamous Cell Carcinoma
8074	Squamous Cell Carcinoma, spindle cell	Squamous Cell Carcinoma
8075	Squamous cell carcinoma, adenoid	Squamous Cell Carcinoma
8076	Squamous cell carcinoma in-situ, possible stroma invasion	Squamous Cell Carcinoma
8078	Squamous cell carcinoma, horn formation	Squamous Cell Carcinoma
8083	Basaloid squamous cell carcinoma	Squamous Cell Carcinoma
8084	Squamous cell carcinoma, clear cell type	Squamous Cell Carcinoma
8032	Spindle cell carcinoma, NOS	Squamous Cell Carcinoma
8140	Adenocarcinoma	Adenocarcinoma
8144	Adenocarcinoma, intestinal type	Adenocarcinoma
8255	Adenocarcinoma with mixed subtypes	Adenocarcinoma
8260	Papillary adenocarcinoma	Adenocarcinoma
8261	Adenocarcinoma in-situ in villous adenoma	Adenocarcinoma
8262	Villous adenocarcinoma	Adenocarcinoma
8210	Adenomatous polyp	Adenocarcinoma
8263	Adenocarcinoma in situ in tubulovillous adenoma	Adenocarcinoma
8310	Clear cell adenocarcinoma	Adenocarcinoma
8313	Clear cell adenocarcinoma fibroma	Adenocarcinoma
8323	Mixed cell adenocarcinoma	Adenocarcinoma
8380	Endometroid adenocarcinoma	Adenocarcinoma
8382	Endometroid adenocarcinoma, secretory variant	Adenocarcinoma
8384	Adenocarcinoma endocervical type	Adenocarcinoma
8430	Mucoepidermoid carcinoma	Adenocarcinoma
8441	Serous adenocarcinoma	Adenocarcinoma
8460	Papillary serous cystadenocarcinoma	Adenocarcinoma
8461	Papillary serous carcinoma	Adenocarcinoma
8480	Mucinous adenocarcinoma	Adenocarcinoma
8481	Mucin producing adenocarcinoma	Adenocarcinoma
8482	Mucinous adenocarcinoma, endocervical type	Adenocarcinoma
8490	Signet ring cell adenocarcinoma	Adenocarcinoma
8560	Adenosquamous cell carcinoma	Adenosquamous
8570	Adenocarcinoma with squamous metaplasia	Adenosquamous
8015	Glassy cell carcinoma	Adenosquamous
8012	Large cell carcinoma	Other
8013	Large cell neuroendocrine carcinoma	Other
8020	Carcinoma, undifferentiated	Other
8021	Carcinoma, anaplastic	Other
8022	Pleomorphic carcinoma	Other
8033	Pseudosarcomatous carcinoma	Other
8041	Small cell carcinoma	Other
8042	Oat cell carcinoma	Other

© 2023 Holt HK et al. *JAMA Network Open.*

8045	Mixed small cell carcinoma	Other
**SEER ICD-O 3 Histology Code**	**Type**	**Histology Classification**
8046	Non-small cell carcinoma	Other
8082	Lymphoepithelial carcinoma	Other
8094	Basosquamous cell carcinoma	Other
8098	Adenoid basal carcinoma	Other
8120	Transitional cell papilloma	Other
8123	Transitional cell carcinoma, spindle cell	Other
8200	Adenoid cystic carcinoma	Other
8201	Cribiform carcinoma	Other
8240	Carcinoid tumor	Other
8244	Composite carcinoid	Other
8246	Neuroendocrine carcinoma	Other
8574	Adenocarcinoma with neuroendocrine	Other
8575	Metaplastic carcinoma	Other
8800	Soft tissue tumor NOS	Other
8801	Spindle cell sarcoma	Other
8802	Giant cell sarcoma	Other
8805	Undifferentiated sarcoma	Other
8806	Small round cell tumor	Other
8896	Myxoid leiomyosarcoma	Other
8900	Rhabdomyosarcoma	Other
8910	Rhabdomyosarcoma, emboyonal	Other
8912	Spindle cell rhabdomyosarcoma	Other
8920	Alveolar rhabdomyosarcoma	Other
8931	Endometrial stromal sarcoma	Other
8933	Adenosarcoma	Other
8935	Stromal tumor	Other
8950	Mullerian mixed tumor	Other
8963	Malignant rhabdoid tumor	Other
8980	Carcinosarcoma	Other
8982	Myoepithelioma, malignant	Other
9064	Intratubular malignant germ cell tumor	Other
9071	Yolk sac tumor	Other
9080	Teratoma	Other
9110	Mesonephric adenocarcinoma	Other
9581	Alveolar soft part sarcoma	Other

Abbreviations: SEER= Surveillance, Epidemiology, and End Results, ICD-O 3=International Classification of Disease for Oncology version 3, NOS=Not otherwise specified

© 2023 Holt HK et al. *JAMA Network Open.*

eTable 2. Exclusion *ICD-O-3* Codes

**SEER ICD-O 3 Histology Code**	**Type**
8500	Intraductal adenocarcinoma
8503	Intraductal papillary adenocarcinoma
8000	Neoplasm, benign
8010	Epithelial tumor
8720	Melanoma
8890	Leiomyoma
8891	Epithelioid leiomyoma
9100	Hydratidiform mole/ Choriocarcinoma
9105	Trophoblastic tumor

Abbreviations: SEER= Surveillance, Epidemiology, and End Results, ICD-O 3=International Classification of Disease for Oncology version 3

© 2023 Holt HK et al. *JAMA Network Open.*

eTable 3. Rates of Treatment With Surgery, Radiation, and Chemotherapy Among Women With Cervical Cancer by Race/Ethnicity and Summary Stage

		Hispanic/Latina (n=5,745)		Non-Hispanic American Indian / Alaskan Native (n=187)		Non-Hispanic Asian / Pacific Islander (n=2,108)		Non-Hispanic Black (n=3,017)		Non-Hispanic White (n=12,435)		
		n	(%)	n	(%)	n	(%)	n	(%)	n	(%)	P*
All Women	Surgery	3,546	(61.7)	111	(59.4)	1,351	(64.1)	1,469	(48.7)	8,007	(64.4)	<0.001
	Radiation	3,063	(53.3)	102	(54.5)	1,090	(51.7)	1,890	(62.6)	6,452	(51.9)	<0.001
	Chemotherapy	2,818	(49.1)	102	(54.5)	1,023	(48.5)	1,707	(56.6)	5,955	(47.9)	<0.001
		**Hispanic/Latina (n=2,966)**		**Non-Hispanic American Indian / Alaskan Native (n=91)**		**Non-Hispanic Asian / Pacific Islander (n=1,052)**		**Non-Hispanic Black (n=1,258)**		**Non-Hispanic White (n=6,631)**		
		n	(%)	n	(%)	n	(%)	n	(%)	n	(%)	P*
Localized	Surgery	2,624	(88.5)	81	(89.0)	973	(92.5)	1,042	(82.8)	6,038	(91.1)	<0.001
	Radiation	722	(24.3)	19	(20.9)	214	(20.3)	417	(33.1)	1,587	(23.9)	<0.001
	Chemotherapy	540	(18.2)	19	(20.9)	158	(15.0)	311	(24.7)	1,156	(17.4)	<0.001
		**Hispanic/Latina (n=2,779)**		**Non-Hispanic American Indian / Alaskan Native (n=96)**		**Non-Hispanic Asian / Pacific Islander (n=1,056)**		**Non-Hispanic Black (n=1,759)**		**Non-Hispanic White (n=5,804)**		
		n	(%)	n	(%)	n	(%)	n	(%)	n	(%)	P*
Regional and Distant	Surgery	922	(33.2)	30	(31.3)	378	(35.8)	427	(24.3)	1,969	(33.9)	<0.001
	Radiation	2,341	(84.2)	83	(86.5)	876	(83.0)	1,473	(83.7)	4,865	(83.8)	0.84
	Chemotherapy	2,278	(82.0)	83	(86.5)	865	(81.9)	1,396	(79.4)	4,799	(82.7)	0.02

*To test for differences between groups we used chi-square test for categorical variables

© 2023 Holt HK et al. *JAMA Network Open.*

eTable 4. Multivariable Product Method Estimates for Proportion of Stage-Related Inequities Mediated by Insurance Status Across Racial/Ethnic Groups

Mediation by commercial insurance versus uninsured		
**Race/ethnicity**	**Percent Mediated (%)**	**(95% CI)**
Hispanic/Latina	51.5%	(49.9, 53.2)
Non-Hispanic American Indian/Alaskan Native	50.8%	(49.9, 51.6)
Non-Hispanic Asian/Pacific Islander	51.2%	(49.9, 52.4)
Non-Hispanic Black	50.3%	(50.0, 50.8)
Non-Hispanic White	Reference	
Mediation by commercial insurance versus Medicaid coverage		
**Race/ethnicity**	**Percent Mediated (%)**	**(95% CI)**
Hispanic/Latina	53.4%	(52.1, 54.7)
Non-Hispanic American Indian/Alaskan Native	51.7%	(51.0, 52.5)
Non-Hispanic Asian/Pacific Islander	52.5%	(51.6, 53.5)
Non-Hispanic Black	50.8%	(50.5, 51.9)
Non-Hispanic White	Reference	

Abbreviations: OR, odds ratio; CI, confidence interval; SES, socioeconomic status
Note: Estimates including area-level SES as an interaction term were similar to our primary approach; results presented here incorporate area-level SES as an interaction variable of the observed racial/ethnic inequities

© 2023 Holt HK et al. *JAMA Network Open.*
