# Supplementary material for: Mediation of Racial and Ethnic Inequities in the Diagnosis of Advanced-Stage Cervical Cancer by Insurance Status
Source: JAMA Netw Open. Author manuscript; Available in PMC 2023 Dec 18. (PMC10726717; doi:10.1001/jamanetworkopen.2023.2985)
Supplement: Supplemental Material 2 — Data Sharing Statement [file NIHMS1943084-supplement-Supplemental_Material_2.pdf]

Data Sharing Statement

Holt. Mediation of Racial and Ethnic Inequities in the Diagnosis of Advanced-Stage Cervical Cancer by Insurance Status. *JAMA Netw Open*. Published March 10, 2023. doi:10.1001/jamanetworkopen.2023.2985

Data
Data available: Yes
Data types: Other (please specify)
Additional Information: Publicly available data
How to access data: Publicly available data
When available: With publication

Supporting Documents
Document types: None

Additional Information
Who can access the data: Publicly available data
Types of analyses: Publicly available data
Mechanisms of data availability: Publicly available data
